# Transcriptome level analysis in Rett syndrome using human samples from different tissues

**DOI:** 10.1186/s13023-018-0857-8

**Published:** 2018-07-11

**Authors:** Stephen Shovlin, Daniela Tropea

**Affiliations:** 1 Neuropsychiatric Genetics Research Group, Trinity Translational Medicine Institute- TTMI, St James Hospital, D8, Dublin, Ireland; 2 Trinity College Institute of Neuroscience, TCIN, Loyd Building, Dublin2, Dublin, Ireland

**Keywords:** Rett syndrome, Methyl-Cpg-binding protein 2, Transcriptomics, MicroArray, RNASeq

## Abstract

The mechanisms of neuro-genetic disorders have been mostly investigated in the brain, however, for some pathologies, transcriptomic analysis in multiple tissues represent an opportunity and a challenge to understand the consequences of the genetic mutation. This is the case for Rett Syndrome (RTT): a neurodevelopmental disorder predominantly affecting females that is characterised by a loss of purposeful movements and language accompanied by gait abnormalities and hand stereotypies. Although the genetic aetiology is largely associated to Methyl CpG binding protein 2 (*MECP2)* mutations, linking the pathophysiology of RTT and its clinical symptoms to direct molecular mechanisms has been difficult.

One approach used to study the consequences of *MECP2* dysfunction in patients, is to perform transcriptomic analysis in tissues derived from RTT patients or Induced Pluripotent Stem cells. The growing affordability and efficiency of this approach has led to a far greater understanding of the complexities of RTT syndrome but is also raised questions about previously held convictions such as the regulatory role of *MECP2*, the effects of different molecular mechanisms in different tissues and role of X Chromosome Inactivation in RTT.

In this review we consider the results of a number of different transcriptomic analyses in different patients-derived preparations to unveil specific trends in differential gene expression across the studies. Although the analyses present limitations- such as the limited sample size- overlaps exist across these studies, and they report dysregulations in three main categories: dendritic connectivity and synapse maturation, mitochondrial dysfunction, and glial cell activity.

These observations have a direct application to the disorder and give insights on the altered mechanisms in RTT, with implications on potential diagnostic criteria and treatments.

## Background

Rett Syndrome (RTT) is a rare (1 in 10,000 live female births) and complex neurodevelopmental disorder frequently associated with mutations in the gene coding for Methl-CpG binding Protein 2 (*MECP2*). Despite the limited genetic aetiology, the clinical presentation of the disorder and the genetic mutations are heterogeneous. The identification of the gene associated to RTT: *MECP2*, lead to the generation of mouse models that recapitulate the signs of the disease. Using the mouse models it has been shown that some of the symptoms of RTT are present even if the gene is regularly expressed in the nervous system (but not the remaining tissues), supporting the observation that RTT is not restricted only to brain malfunctions. This notion stresses the importance of considering several tissues in addition to the brain for the study of RTT.

Changes in gene expression have been explored as an unbiased read out of the molecular mechanisms related to RTT. Our analysis explores the genes dysregulated in different tissues in RTT patients and summarizes the results in accordance with the pathophysiology of the disorder. This is the first review which summarizes the gene expression studies in patients.

### RTT syndrome onset and progression

Rett syndrome is an X-linked, neurodevelopmental disorder found to nearly exclusively affect female patients. Infants have an apparently normal early post-natal development, but signs and symptoms of RTT begin to present around 6 to 18 months after birth. RTT classically is characterised as a loss of purposeful hand movements and acquired skills, loss of language, hand stereotypies such as wringing or clapping and abnormal gait. These symptoms generally progress across stages, including a period of stabilisation after initial regression phase. When the main RTT criteria are present, and accompanied by a period of stabilisation after the regression period, the patients are classified as having typical or classic RTT [1] although there is distinct classification of atypical RTT. Atypical RTT is an umbrella term for a number of RTT variant disorders that exist. Known variants include Early Seizure RTT which is associated with mutations to the *CDKL5* [2], Preserved Speech Variant or Zapella Variant [3], Congenital RTT which is associated with mutations to the *FOXG1* gene [4], “forme fruste” RTT [5, 6] and Male variant RTT [7].

There are four stages of classic RTT: *stage 1* - early onset of disease after birth (between 6 and 18 months), *stage 2 -* developmental regression, *stage 3* - psuedostationary stage (a stabilisation after regression period) and finally *stage 4* - a late motor deterioration stage [8]. Typical RTT is strongly associated with a loss of function mutation to the global transcriptional regulator *MECP2* (*Methyl-CpG-binding protein 2*) and represents over 95% of typical RTT cases and 75% of variant RTT cases [9].

Despite this strong association between *MECP2* mutations and RTT, there are patients with *MECP2* mutations that do not have the clinical presentation- this has been shown to be as high as 1.1% in a north American database of RTT [10, 11]. In atypical RTT -as mentioned previously- other genes such as *CDKL5* and *FOXG1* are associated with the clinical presentation [1]. However recently *CDKL5* has been distanced from RTT as it results in an early onset encephalopathy that tends to present more severely, with greater propensity for epileptic activity [12, 13]. The cases where RTT occurs with no *MECP2* mutations help to illustrate that the genetic aetiology alone cannot confer the RTT phenotype and indeed even within the typical form of RTT there are differing levels of severity between patients. For the purposes of this review we will be specifically looking at studies that use typical RTT patients with the exception of *Colak* et al. *2011* [14] who compared typical RTT and RTT-like patients to control patients to find common pathways between the disorders.

### Methodological approach

Understanding the molecular mechanisms of RTT from transcriptomic analysis of patient derived samples presents several challenges: the rarity of the condition, tissue composition, heterogeneity between samples, and different methods for RNA quantification and analysis.

As RTT is primarily a neurodevelopmental disorder, post-mortem brain samples are the only source that includes tissues with the primary pathology of the disorder and conducting research with them requires age matched, clinically and genetically comparable samples from the same brain regions. The rareness of the disorder make this a tall order. Even if samples are available there is still the problem of heterogeneity of cell populations across brain samples which can bias gene expression [15, 16]. Although this issue has been solved in mouse studies [17], RTT patients present the additional complication that X-inactivation is variable in different cells.

The main dysfunctions of RTT are associated to brain function, yet *MECP2* is a ubiquitously expressed gene across the body and we need to take into account the derivation from different tissues when analysing the results of RNA expression. Recent studies in mice showed that *Mecp2* mutations in whole body but not brain, determine the appearance of symptoms of the disease associated to muscular weakness and bone integrity showing that the brain is not the only area involved in determining the RTT phenotype [18]. These results prompt a re-evaluation of more peripheral tissues for the investigation of molecular dysfunction in RTT, and in particular, the studies performed in peripheral blood, which is a preferred source for studies in patients.

Another interesting aspect to keep in mind when comparing different studies, is the evolving nature of the transcriptomic technology: - RNA-sequencing techniques have replaced microarray as the technique of choice when comparing lowly expressed differential changes. Dynamic range detected with RNA-Seq is optimized when compared to microarray, meaning that resolution of higher fold-changes is improved [19, 20]. The benefits of RNA-Seq arise from the direct reading of the transcript sequences as opposed to microarray which is based on the hybridisation samples to a number of predesigned probes, searching for a limited number of transcripts, developed by manufacturers. Later versions of microarray chips and techniques looked to increase detection of genes by increasing the number of transcripts tested for, with strong replication and reliability of transcript detection [21].

### Post-mortem brain samples

Often considered the gold standard of transcriptomic analysis of neuropsychiatric and neurodegenerative disorders, post-mortem brain samples based studies are understandably rare to come across [22]. The logistical and financial burden of RTT has meant that only 4 transcriptome level studies using post-mortem brain samples [23–26] have been produced. Each of these studies has had to contend with different limiting factors in their approach and analysis meaning that none of the studies are compatible enough to conduct a meta-analysis. Age groups, mutation type, control comparisons, analysis techniques and brain region used all varied across the studies.

*Colantuoni* and *colleagues* provided the first evidence of Differential Gene Expression (DGE) in post-mortem brain (PMB) samples, comparing 6 typical RTT patients with 6 age and sex matched controls across a number microarray platforms and using a number of statistical software programs to increase detection sensitivity and reliability [23]. Brain tissues utilised from the patients were taken from Brodmann areas (BA) 1–5 of the frontal and parietal cortices. Their results identified 3 major areas of DGE: neuronal maturation genes, presynaptic marker genes and glial specific genes. These results point to a disruption of synaptic connections (specifically presynaptically), synapse maturation, synaptic transmission and increased reactivity of glia.

*Deng* and *colleagues* conducted a microarray study to examine DGE in both human RTT patients and murine models. They subsequently used their results of the RTT patient data to generate a mouse model with overexpression of the *FXYD1* gene which was found consistently over expressed in the RTT samples [24]. *FXYD1* is a FXYD domain ion transport regulator which modulates the Na+/K+ ATPase channel activity. *FXYD1* gene over expression in mouse neurons induced reduction in dendrite spine density as determined by Scholl analysis. In support of the role of *FXYD1* gene in RTT, two methylation promotor sites in the *FXYD1* gene have been shown to be binding sites for MeCP2. Further study was carried out on the role of *Fxyd1* in *Mecp2* deficient mice *Matagne and Colleagues* crossed *Fxyd1* null male mice with *Mecp2* ^308^ hetrozygous female mice to examine the behavioural effects of ablating the over expression response of Fxyd1 to *Mecp2* deficiency [27]. The behavioural results of these mice showed a rescue in the recognition of novel location when both alleles for *Fxyd1* were deleted but not with a single allele deletion with no further rescue to any other behavioural testing. They also found that *Fxyd1* KO with normal *Mecp2* expression showed a similar impairment as *Mecp2* deficient mice in this task. These results have shown that modulating the expression of *Fxyd1* levels can rescue very specific hippocampal dependent cognitive functioning.

For *Deng et* al.’s study brain samples were taken from the superior frontal gyrus (SFG) which is adjacent to *Colantuoni* and *colleague’s* samples at BA1–5 (primary somatosensory, primary motor cortex and part of the parietal cortex just posterior to the primary somatosensory cortex). Although these are distinct regions of the cerebral cortex, *MECP2* expression in the human RTT brain is generally distributed across cortical regions although there is a clear preferential expression in matured neurons [15].

In another study *Gibson* et al. *2010* compared frontal and temporal cortices of 6 RTT patients and 6 health sex-matched controls to examine DGE in human RTT Post-Mortem Brian samples. However the lack of age matched in control samples in this study is clearly a confounding factor. In order to compensate for this added level of variability *Gibson* and *colleagues* used four way comparison (Both frontal and occipital cortices of RTT and control samples) to analyse differential expression [25]. For DGE comparison a gene was required to be significantly expressed in 5 out of 6 patients. The idea in this study is to compare a region highly effected in severe RTT phenotypes (Frontal cortex) versus a region thought to be less affected (occipital cortex) as well as the traditional control versus disease comparisons [28, 29]. The results indicated an upregulation of Dynamin 1, Amyloid like protein 1, Clusterin, Cytochrome C Oxidase, and Collapsin Response Mediator Protein 1 (DMN1, APLP1, CLU, MT-CO1, *CRPM1*) in frontal cortex. Interestingly the Clusterin protein appears to be induced by Histone deacetylation inhibitors [30] which would make sense when considering *MECP2*’s molecular mechanism is thought to be mediated by recruitment of Histone Deacetylase 1, HDAC [31]. However recent studies on brain evoked activity in RTT patients showed that the occipital area presents circuits alteration in RTT [32] making assumption of *Gibson and colleagues* weaker.

The final study was conducted by *Lin* and *colleagues* analysed samples from 4 RTT patients and 4 age-, sex- and ethnically matched controls. They used a murine model of RTT to provide further independent verification of the expression of the differentially expressed genes [26]. Two distinct chemistries were used in this study, microarray and RNA-seq. There findings which were confirmed using an independent RTT mouse model dataset [33] and showed 13 significantly differentially expressed genes. Notably the C1Q complex genes *C1QA*, *C1QB* and *C1QC* were all found to be decreased in expression, while a number of complement pathways C3, *TGFBR2*, CXCR1 and TYROBP were also observed to be downregulated.

### Blood tissue samples

As previously alluded, brain samples can be problematic for transcriptomic research in tissues because the samples can only be attained post mortem. This is a serious draw back considering the regressive nature of RTT as well as the distinct stages in classic RTT. On the other hand blood is a tissue that is relatively accessible and is minimally invasive. These properties make blood samples ideal for time course studies taken at multiple times and giving researchers the ability to temporally measure variables in the same RTT patient.

*Pecorelli* and *colleagues* used a Microarray technique to analyse DGE of Peripheral blood lymphomonocytes (PBMC) of 12 RTT patients compared to 7 age and sex matched controls. The study used 2 analysis models to find significant differential expression, these analysis programs were highly congruent 480 differentially expressed genes (DGE) with only 11 genes being found in only one of the programs [34].

Gene Ontology term and clustering analysis was then performed to identify pathways either up or down regulated, showing four major components: genes with chromatin folding were down-regulated, while genes relating to mitochondrial functioning, genes relating to antioxidant defence, and genes with ubiquitin-proteasome system functions were all up-regulated in patients versus controls. The authors suggest a feedback response: expression of antioxidant defence and Ubiquitin Proteasome System (UPS) related genes would increase in response to the altered expression of mitochondrial functioning proteins creating overall an increase in the oxidative stress.

Other evidence has shown that RTT is associated with a higher level of oxidative stress and production of Reactive Oxygen Species (ROS) [35, 36]. *Pecorelli and colleagues* showed upregulation of a number of mitochondrial complex genes in RTT PBMC suggestive of a reduced respiratory efficiency and an aberrant production of ATP levels, although ATP levels were not directly measured in this study. Taken together the upregulation of mitochondrial functioning related genes along with the increase in cellular anti-oxidant defence related genes, it is likely that there was an increased production of ROS. ROS are also known to effect the proper folding and function of proteins, oxidised proteins are dysfunctional and require clearing via the ubiquitin-proteasome related genes [37] by the Ubiquitin-Proteasomal system (UPS). The upregulation of Ubiquitin-proteasome related genes in this study, Pecorelli et al. 2013 is indicative once again of the increased oxidative stress of RTT patients.

*Colak* and *colleagues* used whole blood as their tissue source of 3 typical RTT patients and 2 RTT-like phenotypes and compared them to 7 age and sex matched controls using microarray analysis. The purpose of their work was to identify potential shared mechanisms between typical and atypical RTT. Their data suggested that RTT-like patients have dysregulation in oxidative phosphorylation, mitochondrial functioning, tumour suppressor p53 signalling and docasahexaenoic acid signalling. Of particular note here was the RTT-like patients- but not classic RTT patients- showed a dysregulation of mitochondrial functioning. Network analysis identified potentially critical regulatory functions of the following genes, *IL1, IL1R1*, *TGFβ*, *interferon-α* and *–β* and NFκB pathways in both classic RTT and RTT-like patients. The NFκB pathway is thought to have a critical role in synapse development [38]. As well as NFκB pathway, Calcium homeostasis, cholesterol metabolism and NFAT/Calcinurin signalling were implicated through the bioinformatic analysis in both phenotypes.

### Induced pluripotent stem cells derived from Rett patients

Induced pluripotent stem cells (IPS) are a relatively recent technique that represent an intriguing solution to the inability of researchers to study directly RTT neuronal cells in vivo. IPS cells are cultured from primary cell sources and treated to reverse their cell fate using a number of overexpressed reprogramming factors OCT4, SOX2, KLF4, and MYC. These reprogrammed cells have pluripotency and the ability to be cultured and differentiated into specific cell types depending on culturing microenvironment [39]. Recently these techniques were employed using RTT patient fibroblasts to reprogram and then differentiate IPS cells into RTT phenotypic neurons [40–43]. The findings from these studies showed that the differentiated neuronal cells from cultured from RTT-IPS cells were consistent with RTT phenotype. Interestingly, some studies found that IPS cells and the derived neurons retained non-random highly skewed X Chromosome Inactivation or XCI [43–45] while others have shown a reactivation of the fibroblasts inactive X Chromosomes [40, 41], however, once all IPS cells were cultured and differentiated into neurons, all cells showed XCI.

The RTT-IPS cells with retained XCI can be cultured in such a way as to provide either an isogenic population of IPS derived neurons cultures with either wildtype or mutant *MECP2*. This represents a particularly strong model for examining the effects of XCI on RTT patients. RTT-IPS cells that had full reactivation of both allelic pairs on X Chromosomes is a more representative model for the mosaic expression that occurs naturally in RTT patients. Both models have advantages for researching certain aspects of RTT, but it is important to note that because these studies did not use expression analyses, it is difficult to objectively conclude that the RTT-IPS cells had full or partial XCI [45]. Another consideration is that in IPS cells passaged a lower number of times there is a greater risk to retain residual epigenetic signature from the cell’s original state [46], however to generate isogenic populations of Wildtype and mutant RTT-IPS derived neurons it is more efficient to use lower number passaged [43].

*Tanaka* and *colleagues* completed a recent study investigating DGE in undifferentiated RTT-IPS cells to investigate the regulatory role of *MECP2* loss of function mutations on early cell development. They used both mutant and control *MECP2* expressing cell line from the fibroblasts of RTT patients, sequenced the samples and found that mutant RTT-IPS cells showed de-repression of X-linked genes [47]. Several biological pathways were also found to be affected in mutant RTT-IPS cells but importantly each *MECP2* mutation appeared to give a different pathway profile. Importantly, stem cell development processes did not appear to be affected by *MECP2* mutant expression, although maturation marker NOTCH1 was repressed in all RTT-IPS cell lines and AKT1, another maturation marker was found to be significantly altered in 2 out of 5 mutant RTT-IPS cell lines. Altogether these results show that even at an undifferentiated stage of cell development, RTT cells are beginning to diverge from normal development. The unique profiles shown by each *MECP2* mutation cell line highlight the importance of developing methods for accurate treatment screening for RTT with potential applications in future medical care.

According to the findings in each of these different studies described we identified three main mechanisms that are altered in RTT: dendritic arborisation and synaptic maturation, mitochondrial function, and glial activity. In the following sections we will describe these in more detail and how other work in the literature backs up these mechanisms.

### Abnormalities in dendritic Arborisation and synaptic maturation

Although the first real signs of the onset of RTT are the impaired developmental and neurocognitive symptoms that present between 6 months and 1 year [8], in some patients microcephaly has provided an even earlier indication of the presence of RTT [48]. Brain weight and volume too have been shown to be decreased in early post-mortem analysis of RTT [29, 49]. These Early studies have indicated that RTT brain morphology may be drastically different to warrant such global changes.

Indeed when the microstructure of RTT neurons from human patients and mouse model RTT brains was analysed, decreases in dendritic spine density and neuronal cell soma size were repeatedly observed [49–52]. These two characteristics, decreased dendritic spine density and neuronal soma size are thought to be the salient morphological changes that occur in the RTT brain phenotypes [53, 54]. This phenotype has been linked to RTT genetically by the theory that loss of function mutations in *MECP2* disrupt synaptic maturation processes at a critical time in development, causing deficient dendritic expansion, an increase in *BDNF* and neurotransmitter abnormalities which would all contribute to further dendritic reductions and synaptic pruning [55].

Given this background, it comes as no surprise that the transcriptomic studies in human post-mortem brain samples provided strong evidence to support the abnormalities to dendritic spine dysgenesis and synaptic maturation. There was a decrease in presynaptic markers found in *Colantuoni and colleagues study* as well as some increase in the postsynaptic markers [23]*,* while *Deng* and *colleagues* found that *FXDY1* increased expression in both human and mice brain samples and resulted in decreased synaptic density when *Fxdy1* was overexpressed in murine models. *Gibson and colleagues* found that *CRMP1,* which is normally localised in the dendrites of hippocampal neurons and is involved in neural process outgrowth, showed an increase in the frontal cortex. They hypothesised that abnormal expression of *CRMP1* could contribute to a decrease in dendritic arborisation, through abnormal process outgrowth and Long-term potentiation [25]*. Colak* and *colleagues* results in whole blood show that the NF-kB (Nuclear Factor Kappa B Subunit 1) pathway was observed to be disrupted in whole blood of RTT and RTT-like patients. Increased NF-kB signalling has been shown to contribute to loss of dendritic spine density at the callosal projection neurons of *MeCP2* knockout mice. Decreasing this aberrant NF-kB signalling could rescue dendritic phenotype and improved the survival of such KO mice [56]. Specifically *Colak and colleagues* found that NFAT complex genes: *NFATC2* and *NFATC3* were downregulated, and postulate that this disruption to the NFAT/Calcineurin complex caused a decrease to axonodendritic connections and disrupted synaptic proliferation in both RTT and RTT-like blood tissue [14].

The bulk of the evidence for the disruption of synaptic maturation was shown by *Colantuoni and colleagues* who found increases to excitatory neurotransmitter receptors (APMA1 and AMPA2) genes and decreases to inhibitory neurotransmitters receptors (GABRB3) genes were occurring in frontal and parietal lobes of the cerebral cortices of RTT patients. Neurotransmitter imbalances has been thought to be responsible for the loss of neuronal function in RTT patients [55]. On top of this there was also decreases to a number of specific maturation markers such as neuron specific enolase MAP2, Tau, and synaptic vesicle proteins, SNAP25, DOC2A, syntaxin and annexin, which have been used to measure clinical neuropathologies [57]. As mentioned before this inability for normal synaptic maturation from the loss of *MECP2* expression is thought occur at a critical time in development that effects neuronal action and membrane properties [55, 58]. However the effects of the decreased synaptic maturation appears to occur post transcriptionally making it difficult for microarray studies to detect [59]. *Tanaka and colleagues* found that undifferentiated RTT-IPSC showed overrepresentation of synaptic transmission, axon guidance and neural projection development which would indicate that changes take place earlier in development. These pathways were not equally altered across patients and indicated that each *MECP2* mutation had a different profile of disrupted pathways [47].

Not all evidence from the transcriptomic studies pointed directly to the loss of dendritic arborisation and loss of synaptic maturation though. *Lin and colleague’s* major finding in RTT Post-mortem brains was a downregulation of the C1Q complex genes. These genes play a role in microglial synaptic pruning. Downregulation of C1Q is suggestive of less synaptic pruning and therefore an increased dendritic spine density however this is no direct measure of dendritic spine density in this study [26].

Although it is not possible to state definitively that the loss of dendritic arborisation is the primary change responsible for clinical manifestations the RTT phenotype, it is clear the recurrence of these structural abnormalities likely contribute to the RTT phenotype or at least the neurocognitive deficiencies. This hypothesis is supported by recent work by *Ross and colleagues* [18] who explored the peripheral component of RTT, by creating a Peripheral *Mecp2* Knockout (PKO) mouse model which specifically addresses the effects of mice with neurotypical architecture but disrupted *Mecp2* expression in the rest if the body. Their findings showed much improved severity score, survival, and body weight, although after a year wildtype and PKO were still significantly different weights. The study showed that the central nervous system component of RTT is responsible for the majority of the symptoms of RTT. Behavioural, sensorimotor and even autonomic deficiencies appeared to be rescued in these mice. However a peripheral phenotype was observed, hypo-activity, exercise fatigue and bone abnormalities were all detected in the PKO mice. The importance of this finding should not be over looked, hypo-activity and fatigue are interesting in the context of *MECP2*’s effect on mitochondrial dysfunction which will be discussed in the subsequent section.

There are two main outcomes of these studies: first, results derived from IPSCs are not totally overlapping with results coming from brain samples. This is likely to be due to the different stage of maturation in the samples from the two different populations. Second, although- as expected- the majority of the genes dysregulated in the category of dendritic connectivity and synaptic maturation derive from studies on brain post-mortem samples, some genes identified in blood samples are also been showed to be involved in synaptic maturation (i.e. *NFkB pathway*).

A list of the genes that evidence the disruption to synaptic maturity and dendritic arborisation were detected as significantly different in across these studies is listed below in Table 1.

**Table 1:** Summary of the gene expression evidence for changes in dendritic arborisation and synaptic maturation identified by transcriptomic analyses in human Rett Syndrome tissues

Dendritic Arborisation and Synaptic Maturation					
Gene	Expression	Tissue	Gene	Expression	Tissue
*FXDY1* ^*2*^	↑	FC	*SYT5* ^***1***^	↓	FC & PC
*EAAT2* ^*2*^	↑	FC	*VAMP2* ^***1***^	↓	FC & PC
*NTRK2* ^*2*^	↑	FC	*NSF* ^***1***^	↓	FC & PC
*ANXA6* ^*1*^	↓	FC & PC	*NPTX1* ^***1***^	↓	FC & PC
*STX1A* ^*1*^	↓	FC & PC	*PPP3CB* ^***1***^	↓	FC & PC
*DOC2A* ^*1*^	↓	FC & PC	*GABRD* ^***1***^	↓	FC & PC
*SNAP-25* ^*1*^	↓	FC & PC	*GRIN2A* ^***1***^	↓	FC & PC
*14–3-3 β/τ/ζ/ν* ^*1*^	↓	FC & PC	*CAMK2B* ^***1***^	↓	FC & PC
*MAP2* ^*1*^	↓	FC & PC	*NOEL* ^***1***^	↓	FC & PC
*MAP1A* ^*1*^	↓	FC & PC	*NNAT* ^***1***^	↓	FC & PC
*MAPT* ^*1*^	↓	FC & PC	*ENO2* ^***1***^	↓	FC & PC
*GRIA1* ^*1*^	↓	FC & PC	*PCP4* ^***1***^	↓	FC & PC
*GRIA2* ^*1*^	↓	FC & PC	*INA* ^***1***^	↓	FC & PC
*GABRB3* ^*1*^	↑	FC & PC	*NEFH* ^***1***^	↓	FC & PC
*GRIN1* ^*1*^	↑	FC& PC	*NFATC2* ^*6*^	↓	WB
*GRM1* ^*1*^	↑	FC & PC	*NFATC3* ^*6*^	↓	WB
*GRM8* ^*1*^	↑	FC & PC	*CRMP1* ^*3*^	↑	FC
*GABRR1* ^*1*^	↑	FC & PC	*DNMI* ^*3*^	↑	FC
*GABRP* ^*1*^	↑	FC & PC			
*SYP* ^*1*^	↓	FC & PC			
*SYN2* ^*1*^	↓	FC & PC			
*SYNGR1* ^*1*^	↓	FC & PC			
*SYNGR3* ^*1*^	↓	FC & PC			
*SYT1* ^*1*^	↓	FC & PC			

Colantuoni et al. [23]^1^, Deng et al. [24]^2^, Gibson et al. [25]^3^, Lin et al. [26]^4^, Pecorelli et al. [34]^5^, Colak et al. [14]^6^, Tanaka et al. [47]^7^. *FC* Frontal Cortex, *PC* Parietal Cortex *TC* Temporal Cortex, *WB* Whole Blood, *PBMC* Peripheral Blood Lymphmonocytes, *RTT-IPS* Rett patient derived Induced pluritpotent Stem cells

### Mitochondrial dysfunction

The link between RTT and mitochondrial dysfunction was made by a number of researchers back in the early 1990s [60–62]. The rationale behind these studies was the overlap in symptoms found in RTT and in mitochondrial disease: early developmental delay, mental retardation, seizures, motor dysfunction, GI reflux, cardio- and respiratory problems [63]. Mitochondrial dysfunction and diseases are associated with an increase in oxidative stress due to imbalance in energy production which leads to the generation of ROS [36, 64, 65]. Mitochondrial dysfunction has been less studied as a mechanic of the pathophysiology of RTT compared to the neuronal and developmental mechanisms but has recently received more attention. *Kriaucionis and colleagues* [66] reopened the question of mitochondrial dysfunction in RTT with the discovery *Ubiquinol-cytochrome c reductase core protein 1* or *Uqcrc1* overexpression in RTT mouse model. Since then findings in the relationship between mitochondria dysfunction and RTT phenotype have been gathering [67–69], this has culminated in the EPI-743 phase II clinical trial in 2014 (NCT01822249) which is a vitamin E compound targeting energy production via targeting of NADPH quinone oxidoreductase 1 (NQO1) developed by Edison pharmaceuticals. A more recent vitamin E derivative Trolox has been developed and has recently been tested in preclinical models [70].

Evidence for disturbed mitochondrial function in RTT was predominantly derived from Pecorelli et al. 2013 [34] study in human RTT whole blood. With their findings implicating mitochondrial complexes 1 to 5 as well as ATP synthase and ATPase inhibitory factor gene 1 all upregulated in RTT whole blood. Of particular note is the *Cytocrome C Oxidase/COX* genes which were upregulated with a mean fold increase of 1.5 times. COX expression and enzymatic activity were examined in the frontal cortex of post-mortem RTT brains in *Gibson* et al.*’s* [25] work, where a reduction in both of these was observed. Altered *Cytochrome b-c 1 complex subunit 1* or *UQCRC1* expression had previously been observed by *Kriancious* et al. and shown to be disrupt mitochondrial respiration in mouse neuroblastoma cell culture [66]. This paper also supports the observations that mitochondrial complexes I and III are down and upregulated respectively in RTT.

*Colak and Colleagues* [14] found that only RTT-like patients had mitochondrial dysfunctions through network analysis of whole blood samples, where classic RTT did not show the same extent of mitochondrial dysfunction relative to the RTT-like patients. However there was a very limited number of patients used it in this samples that would affect the statistical power of the experiment (3 classic RTT and 2 RTT-like samples were used by *Colak and colleagues* compared to 12 classic RTT samples in *Pecorelli and colleagues* study in 2013 paper [14, 34]). There is further evidence for and against mitochondrial dysfunction mechanism in the in RTT derived Induced Pluripotent Stem cells (RTT-IPS). In undifferentiated RTT-IPS there was an increase in expression of *NR3C1*, which encodes a mitochondrial transcription factor as well as MRPS33 which encodes a mitochondrial ribosomal protein. *MRPS33* was also increased in the PBML while *NR3C1* was not differentially expressed. However when RTT-IPS were differentiated towards neural cell fate in Andoh-Noda and Colleagues work [71], the increase to *NR3C1* was not found. Interestingly, genes linked to mitochondrial dysfunction appear to be dysregulated in all the cells and tissues examined. The list of genes supporting the mitochondrial dysfunction observed in RTT human tissues comparted to control can be found listed below in Table 2.

**Table 2:** Summary of the gene expression evidence for changes in mitochondrial functioning genes identified by transcriptomic analyses in human Rett Syndrome tissues

Mitochondrial Functioning Genes					
Gene	Expression	Tissue	Gene	Expression	Tissue
*COX6C* ^*5*^	↑	PBMC	*COX6C* ^*5*^	↑	PBMC
*COX7A12* ^*5*^	↑	PBMC	*ETFA* ^*5*^	↑	PBMC
*COX7C* ^*5*^	↑	PBMC	*UQCRQ* ^*5*^	↑	PBMC
*COX8A* ^*5*^	↑	PBMC	*TIMM10* ^*5*^	↑	PBMC
*COX14* ^*5*^	↑	PBMC	*TSPO* ^*5*^	↑	PBMC
*URCRQ* ^*5*^	↑	PBMC	*TOMM7* ^*5*^	↑	PBMC
*UQCRFS1* ^*5*^	↑	PBMC	*MT-CO1* ^*3*^	↓	FC
*UQCRH* ^*5*^	↑	PBMC	*MRPS33* ^*5,7*^	↑	RTT-IPS & PBMC
*SDHB* ^*5*^	↑	PBMC	*NR3C1* ^*7*^	↑	RTT-IPS
*NDUFV2* ^*5*^	↑	PBMC			
*NDUFS4* ^*5*^	↑	PBMC			
*NDUFA9* ^*5*^	↑	PBMC			
*NDUFS6* ^*5*^	↑	PBMC			
*NDUFB10* ^*5*^	↑	PBMC			
*NDUFB4* ^*5*^	↑	PBMC			
*NDUFC2* ^*5*^	↑	PBMC			
*NDUFS5* ^*5*^	↑	PBMC			
*NDUFC1* ^*5*^	↑	PBMC			
*NDUFB9* ^*5*^	↑	PBMC			
*NDUFA8* ^*5*^	↑	PBMC			
*NDUFAB1* ^*5*^	↑	PBMC			
*NDUFA2* ^*5*^	↑	PBMC			
*NDUFB6* ^*5*^	↑	PBMC			
*ATP5A1* ^*5*^	↑	PBMC			

Colantuoni et al. [23]^1^, Deng et al. [24]^2^, Gibson et al. [25]^3^, Lin et al. [26]^4^, Pecorelli et al. [34]^5^, Colak et al. [14], Tanaka et al. [47]^7^. *FC* Frontal Cortex, *PC* Parietal Cortex *TC* Temporal Cortex, *WB* Whole Blood, *PBMC* Peripheral Blood Lymphmonocytes, *RTT-IPS* Rett patient derived Induced pluritpotent Stem cells

A further point of interest here is that two studies have discussed both mitochondrial dysfunction and dendritic abnormality together. *Großer* and *colleagues* postulated how mitochondrial dysfunction in RTT could affect dendritic signal integration and plasticity while *Belichenko* and *colleagues* went as far as to identify not only coincidence but show co-localisation of both dendritic spine dysgenesis and mitochondrial dysregulation in mutant *Mecp2* mice [51, 72]. Specifically they found enlargement of mitochondria and altered structure of cristae at the dendrites of mouse neurons. Recent attempts to target the mitochondrial dysfunction in RTT has been strong enough to warrant human testing with clinical trials like the EPI-743 and Triheptanoin phase 2 trials (NCT02696044 and NCT01822249 respectively). These trials could determine the utility of targeting mitochondrial dysfunction in RTT. Considering all this evidence and the real possibility of clinical application in a mitochondrial rescue approach to treating RTT patients, mitochondrial dysfunction represents an important feature of Rett that is supported by a number of the transcriptomic studies.

### Glial cell activity

In recent times the role of glial cells has been explored in RTT patients. Initially glial cells were thought not to be important in the study of RTT as *MECP2* was considered exclusively neuronally expressed in the CNS. Earlier immunocytochemical methodologies were unable to detect glial *MECP2* expression in humans [15] and therefore the role of glia was diminished in RTT. However more recent research conducted in the role of glia and RTT has yielded more contradictory results. Microglia have been found to be partly responsible for or at least contribute the RTT phenotype in mouse models [73] in addition, astrocytic re-expression of *MECP2* has also been observed to improve motor and respiratory deficits and increase longevity in mice models of RTT [74]. However reintroduction of Wildtype microglia has been shown not to be able to rescue normal functioning [75]. These findings in RTT mouse models were foreshadowed though by the *Colantuoni and colleagues* [23] whose transcriptomic analysis of RTT patient’s post-mortem brain samples detected increases in a number of specific glial cell markers including *GFAP, S100A13, α B-crystallin,* and *EAAT1*. This insight into observed glial expression that occurred directly in the brain tissues of RTT patient’s years before it was observed in preclinical models. This illustrates the value of measuring gene expression in human patients in spite of the technical and logistical challenges of such experiments present.

Contrary to the previous attempts to quantify *MECP2* expression in glia, more sensitive antibodies have made it possible to detect *MECP2* expression in astrocytes. Non-cell autonomous impact from *MECP2* mutant astrocytes has been shown to perpetuate a RTT like phenotype too [74, 76, 77]. These detrimental effects could also be rescued via appropriate expression of *MECP2* in the effected astrocytes [74]. Glutamate has been strongly implicated as being the major player in this non-cell autonomous effect, specifically glutamate clearance [78, 79]. This is especially interesting considering the findings from *Colantuoni* et al. and *Deng* et al. respectively [23, 24] who the glutamate transporters *EAAT1* and *EAAT2* are both upregulated in the RTT human brain. *EAAT2* or *Glutamate transporter 1* is also found in high concentrations on normal astrocytes in throughout the brain and is responsible for 90% of all glutamate uptake [80] while *EAAT1* or the *glutamate aspartate transporter* is expressed earlier in development and expressed in lower concentration than *EAAT2* [81].

Downregulation of *EAAT1* and *EAAT2* expression in response to glutamate exposure have been shown to be impaired in astrocytic cultures of *MECP2* KO mice compared to wildtype, illustrating an acceleration of Glutamate clearance [79]. On top of this abnormal glutamate metabolism, microglia have shown an increased release of glutamate, which is thought to contribute the aberrant dendritic architecture in neurons [78]. The proteins produced by *GFAP* and *S100β* are both increased in astrocytes of *Mecp2* KO mice [79] which again were found to be increased in post-mortem RTT frontal cortices [23, 24].

Further evidence of disruption to microglial was also found across the transcriptional studies. *Clusterin* or *APO-J* produces a protein associated with microglial activation [82] was increased in post-mortem RTT brain in *Gibson* et al.*’s study*. *Lin and colleagues* found that C1Q complement genes were downregulated in RTT human brains, they postulated three possibilities. Either there was a reduction in the total number of microglia, or there was normal resting state microglia but a reduced activation, or that the decreased expression of C1Q was found in neurons [26]. Table 3 lists the genes differentially expressed across the transcriptomic studies that support the role of glial cell activity in RTT pathology.

**Table 3:** Summary of the gene expression evidence for changes in glial cell activity identified by transcriptomic analyses in human Rett Syndrome tissues

Glial Cell Activity					
Gene	Expression	Tissue	Gene	Expression	Tissue
*FGFR3* ^*2*^	↑	FC	*APO-E* ^*1*^	↑	FC & PC
*EAAT2* ^*2*^	↑	FC	*APO-J* ^*3*^	↑	FC & PC
*S100 β* ^*2*^	↑	FC	*EAAT1* ^*1*^	↑	FC & PC
*C1QA* ^*4*^	↓	FC & TC	*CRYAB* ^*1*^	↑	FC & PC
*C1QB* ^*4*^	↓	FC &TC	*GFAP* ^*1*^	↑	FC & PC
*C1QC* ^*4*^	↓	FC & TC	*S100A13* ^*1*^	↑	FC & PC

Colantuoni et al. [23]^1^, Deng et al. [24]^2^, Gibson et al. [25]^3^, Lin et al. [26]^4^, Pecorelli et al. [34]^5^, Colak et al. [14]^6^, Tanaka et al. [47]^7^. *FC* Frontal Cortex, *PC* Parietal Cortex *TC* Temporal Cortex, *WB* Whole Blood, *PBMC* Peripheral Blood Lymphmonocytes, *RTT-IPS* Rett patient derived Induced pluritpotent Stem cells

In *Colak* and *colleagues* study comparing the gene expression between RTT and RTT-like patient’s whole blood the Interleukin-4 pathways were altered in both patient types. IL-4 is a cytokine responsible for M2 phagocytic clearance in the brain, where it helps to switch microglia to a polarised M2 expressing cells, which optimises the microglia to provide neuroprotective functions. These functions include releasing neurotrophic factors and clearing the ischemic debris which can be caused by neuroinflamatory processes [83]. Considering the evidence that RTT patients have altered inflammatory responses [84, 85] the IL-4 pathway dysregulation could indicate that this impaired microglial switch in RTT patients could contribute to the pathogenic role of Microglia and neuroinflammatory processes in the disease.

The RTT-Induced Pluripotent Stem cells have been studied by *Andoh-Noda and colleagues,* and they found that *MECP2* mutations found in RTT patients caused an increase of expression of glial markers in differentiated cell cultures suggesting a bias towards a astrocytic cell fate [71]. These results help to show the contribution of glial cell activity towards disruption of the synaptic transmission, dendritic and synaptic architecture in the brains of human RTT brains.

The role of glial cells and their contribution to the RTT phenotype certainly has changed over recent years but there are still many unanswered questions with regards to the potential therapeutic targeting of this mechanism [86]. However given the amount of evidence and indications from the transcriptomic studies as well as the current directions of the literature, it is likely that glial function will be a focus in the future of RTT research, also considering that several of the reported genes have been identified in both brain, whole blood and IPSCs and are linked to mechanisms of inflammation which are known to be present in multiple tissues.

### Influence of mutation type on mechanisms:

As well as the heterogeneity of tissue type, one interesting aspect that might contribute to lack of overlapping in these transcriptomic studies is how mutation type contributes to the severity of the disorder. Various studies have found a number of links between particular mutations and severity of disease [10, 87–89]. This has been an area of great interest to clinicians and researchers, as finding relationships between molecular mechanisms of MECP2 and severity of phenotype could elucidate possible therapeutic targets and/or provide genetic counselling for families and carers. Since 99.5% of RTT patients have De Novo mutations [90] occurring in different portions of the *MECP2* gene, the composition of RTT patients examined in each cohort will likely vary, hence influencing the differential gene expression detected throughout these studies.

The scope of individual mutations in the *MECP2* is large, recently it was found that 518 different pathogenic or likely pathogenic mutations have been recorded while a further 211 mutations’ significance has yet to be determined out to the approximately 900 different recorded cases of *MECP2* mutation [91]*.* Despite this wide range of variance > 65% of RTT syndrome mutations are caused by a subset of 8 common mutations; R106W, R133C, T158 M, R168C, R255X, R270X, R294X, and R306C [92]. Interestingly, this distribution is reflected in the total cohort of patients across these transcriptomic studies, 19/29 = 65.5% (see. Table 4).

**Table 4:** Displaying the mutation classification of each RTT patient in the individual studies. Sample identifier number, age, genetic mutation, amino acid change and effected domain are all detailed

Study	Sample number	Age (Years)	Mutation gene	Amino Acid change	Effected Domain
*Post-Mortem Brain Studies*					
Colantuoni et al. 2001 [23]	RTT1	2	c.502 C > T	p.R168X	ID
	RTT2	10	c.808 C > T	p.R270X	TRD-NLS
	RTT3	25	c.880 C > T	p.R294X	TRD
	RTT4	29	No coding region mutation detected	NA	
	RTT5	19	c.880 C > T	p.R294X	TRD
	RTT6	19	No coding region mutation detected	NA	
Gibson et al. 2010 [25]	RTT1	11	c.763 C > T	p.R255X	TRD-NLS
	RTT3	12	c.808 C > T	p.R270X	TRD-NLS
	RTT4	18	c.473 C > T	p.T158 M	MBD
	RTT5	11	c.316C > T	p.R106W	MBD
	RTT6	21	c.808C > T	p.R270X	TRD-NLS
	RTT9	4	c.750insC	p.P251fs	TRD
Lin et al. 2016 [26]	RTT1	10	c.378-2A > G	splice site	Intronic region
	RTT2	9	c.763 C > T	p.R255X	TRD-NLS
	RTT3	7	c.451G > T	p.D151Y	CTD
Deng et al. 2007 [24]	Data not available				
*Blood Studies*					
Colak et al. 2011 [14]	Data not available				
Pecorelli et al. 2013 [34]	RTT1	7	Early truncating mutation	NA	
	RTT2	10	c.403A > G	p.K135E	MBD
	RTT3	9	c.403A > G	p.K135E	MBD
	RTT4	9	c.455C > G	p.P152R	MBD
	RTT5	12	c.473C > T	p.T158 M	MBD
	RTT6	19	c.763C > T	p.R255X	TRD-NLS
	RTT7	22	c.806_807delG	p.G268 fs	TRD-NLS
	RTT8	7	c.808C > T	p.R270X	TRD-NLS
	RTT9	7	c.808C > T	p.R270X	TRD-NLS
	RTT10	12	C880C > T	p.R294X	TRD
	RTT11	6	c.880C > T	p.R294X	TRD
	RTT12	11	c.916C > T	p.R306C	TDR
*Induced pluripotent stem cell studies*					
Tanaka et al.2014 [47]	RTT1	5	c.473 C > T	T158 M	MBD
	RTT2	5	c.703C > T	Q244X	TRD
	RTT3	25	c.705delG	E235fs	TRD/CTD
	RTT4	8	c.916C > T	R306C	TDR
	RTT5	5	c. 1461A > G	X487W	CTD

*MBD* Methyl Binding Domain, *ID* Inter Domain, *TRD* Transcriptional Repressor Domain, *TRD-NLS* Transcriptional Repressor Domain-Nuclear Localisation Signal, *CTD* C-Terminal Domain

Even within this subset there are differences in severity and disease progression. *Cuddapah and Colleagues* used the largest cohort of RTT patients to date (1052 participants) and found that mutations to R133C, R294X, R306C, exon 1, and 3′ truncations had lower severity scores while mutations R106W, R168X, R255X, R270X, splice sites, large deletions, insertions and deletions, were all found to have higher clinical severity scores; finally the common mutation T158 M was found to represent an intermediate clinical severity score [89]. It was also found that although –in general- clinical severity increases overtime, this was not true for a number of mutations including R106W, R294X, exon 1 insertions, large deletions, splice sites, and cases without *MECP2* mutations. Considering the progressive nature of RTT these exceptions are intriguing.

In order to better understand the influence of the different genetic mutations in each of these transcriptomic studies, we listed each mutation for each RTT patient in Table 4. However we were not able to access the genotypic information for some studies [14, 24]. Table 4 is split into each group of tissue type, i.e. Post-Mortem Brian studies, Blood Tissue studies and Induced Pluripotent Stem Cells.

**Fig. 1:**
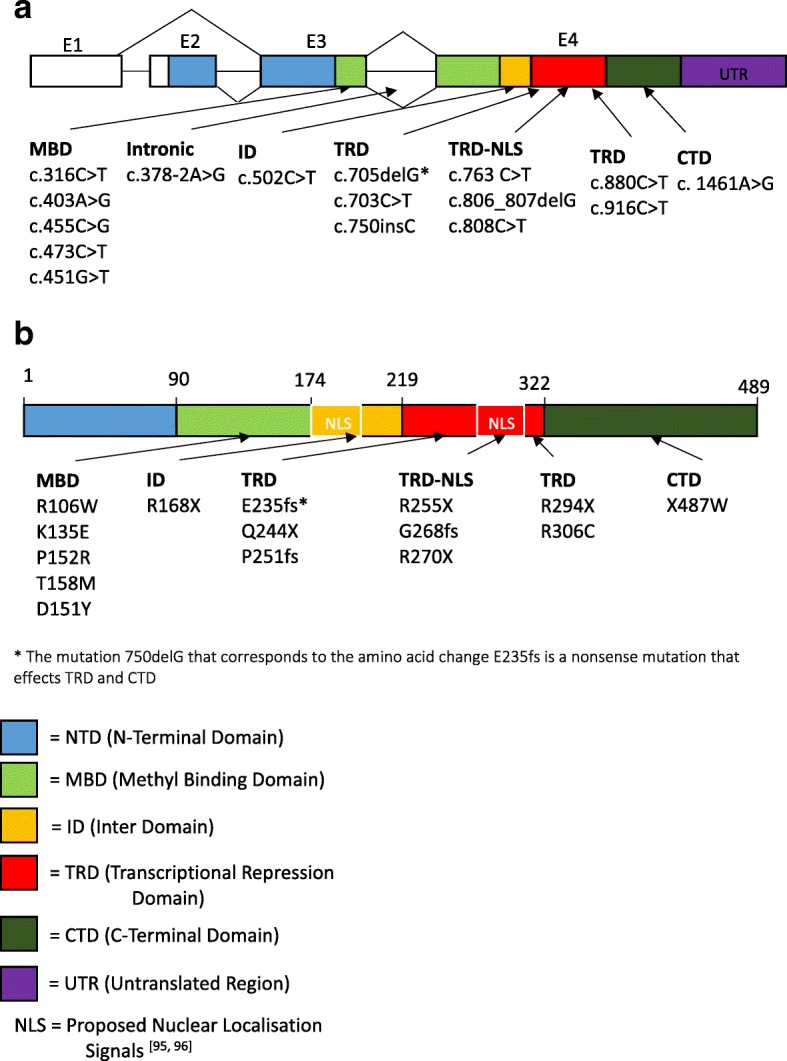
Schematic of the gene structure and protein structure of MECP2. Gene structure (**a**) and protein structure (**b**) annotated with the mutations present from the different transcriptomic studies. Legend: Methyl Binding Domain *MBD*, Transcription Repression Domain *TRD*, Nuclear localisation Signal *NLS*, C-Terminal Domain *CTD* and other including Intronic and splice site

In order to visualize the distribution of the different mutations in the *MECP2* gene, we report the schematics of the different exons and protein domains of *MECP2* gene (Fig. 1a) and protein (Fig. 1b). The mutations found across the studies are grouped as per protein domain and are listed out and displayed on the schematic.

Early work on the structure of MECP2 elucidated 2 well conserved regions: the Methyl Binding Domain (MBD) - an 85 base pair sequence that facilitates the binding of the protein to DNA methyl CpG sites [93], and the Transcriptional Repressor Domain (TRD) -where the protein interacts with transcriptional factors to affect the transcriptional repression once bound to the methylated CpG by the MBD [94]. Further research into the chromatin localisation of MECP2 identified 2 possible regions as being responsible for the localisation of the protein into the nucleus of the cell thus mediating the entire action of the protein. This so called Nuclear Localisation Signal (NLS) has been proposed to be located at 2 different site as shown in  Fig. 1b at amino acid 251–270 [95] and amino acid 173–193 [96]. Work by *Kifayathullah and colleagues* would indicate the 251–270 NLS is not essential to localisation as they found their transgenic mouse line with *Mecp2*^*270*^ localised in the nucleus of both astrocytes and neurons. They postulate that the R270 NLS region may not be critical to nuclear localisation and other NLS are sufficient for the localisation [97]. More recently nuclear localisation has thought to be facilitated by transporter proteins, KPNA3 and KPNA4 in fact KPNA3 binding to MECP2 has been shown to be retained in MeCP2-270X N2a cell lines [98].

Interestingly *Fabio and Colleagues* demonstrated that when RTT patients were split into mutations within NLS (mutations within R294, excluding R294 itself) and mutations after NLS (mutations including R294 onwards through C terminal) they found a significant decrease in severity of Motor function and autonomy impairments indicating its importance in the overall function of the *MECP2* activity [99]. Indeed others too have found evidence that mutations towards the C-terminal of *MECP2* have milder phenotypes. RTT patients with R306C and C-terminal truncations were both identified as being more likely to retain ambulation and use of language [88, 100].

In Table 5 below we show a breakdown of individual mutations identified in studies supporting the 3 mechanisms previously discussed; abnormal dendritic arbours and synaptic maturation, mitochondrial dysfunction and glial cell activation. The two most common mutations were R270X and R294X with 11 and 8 cases respectively. No clear pattern was seen indicating that particular mutations were more likely to support a particular mechanism. Although mitochondrial dysfunction was somewhat higher than the others across domains, this was probably due to *Pecorelli and Colleagues* having a greater study participation (*n* = 12) [34] compared to the other studies [14, 23–26, 47].

**Table 5:** Displaying each RTT patient mutation used across the different studies along with the effected domain. These mutations are split into the 3 mechanisms found across the studies, abnormal dendritic arbours and synaptic maturity, mitochondrial dysfunction and glial activation

Mutation	Domain	Abnormal Dendritic Arbours and Synaptic Maturity	Mitochondrial Dysfunction	Glial Activation	Total
R106W	MBD	X	X	X	3
K135E	MBD		XX		2
P152R	MBD		X		1
T158 M	MBD	XX	XXX	X	6
D151Y	MBD			X	1
Splice site	Intronic			X	1
R168X	ID	X	X	X	3
E235fs	TRD-CTD	X	X		2
Q244X	TRD	X	X		2
P251fs	TRD	X	X	X	3
R255X	TRD-NLS	X	XX	XX	5
G268 fs	TRD-NLS		X		1
R270X	TRD-NLS	XXX	XXXXX	XXX	11
R294X	TRD	XX	XXXX	XX	8
R306C	TRD	X	XX		3
X487W	CTD	X	X		2
No Mutation Detected		XX	XX	XX	4
Total MBD		3	7	3	13
Total TDR		11	17	8	36
Total NLS		4	8	5	17
Total CTD		2	2	0	4
Total Other (ID, intronic)		1	1	2	4
Mechanism total		21	35	18	

*MBD* Methyl Binding Domain, *TRD* Transcription Repression Domain, *NLS* Nuclear localisation Signal, C-Terminal Domain *CTD* and other including Intronic and splice site

One study that took into account individual RTT mutations was the RTT-IPS cell study by *Tanaka* and *Colleagues* who used fibroblasts to create Induced pluripotent stem cells with mutant *MECP2* expressing cell lines, which were then used for sequencing. Their results showed that a number of neurodevelopmental functions were affected, although these varied depending on the mutation. For examples the R306C mutation cell line showed that downregulated genes compared to wild type effected the axonal guidance and neuronal projection but not synaptic transmission, while for E235fs mutations was the opposite, with synaptic transmission effected but not axonal guidance or neuronal projection. And finally the C-terminal deletion X487W was only significantly effected in axonal guidance KEGG pathway. *Tanaka and Colleagues* conclude that even from early development individual *MECP2* mutations affect different sets of genes [47]. Due to the limited number of patients reported in the mentioned transcriptomic studies, we cannot run an association analysis between mutation type and molecular function, however all together these results show that the majority of mutations present are associated to alterations in genes associated to mitochondrial function.

## Conclusion

In summary, despite the limited number of transcriptomic level studies conducted in human RTT patients, there is a small reservoir that provide interesting information for understanding some of the pathophysiology of RTT. The main conclusion that emerges from the human transcriptomic studies, is the convergence of mechanisms across different tissues. The dysregulated genes belong to three main categories: abnormal dendritic arborisation and synaptic maturation, mitochondrial dysfunction and glial cell activity. Analysis in each of these groups lead to new potential therapeutics: clinical trials have been designed utilising compounds to target both the abnormal dendritic architecture (NCT01777542 and NCT01703533) and the mitochondrial dysfunction (NCT01822249 and NCT02696044) in RTT and have reached phase II trials. The evidence for targeting glial cell expression is slightly more contested, however now exists a strong base of evidence to support the disruption to normal function of glial cells including a number of the transcriptomic studies reviewed here (including *Colantuoni, Deng, Lin, Gibson, Colak* and *colleagues* [14, 23–26]).

Although additional analysis is required to confirm the exact pathophysiological events taking place in RTT patients, transcriptomic studies represent a very good unbiased basis for detection of aberrant cellular behaviours and provide researchers with a road-map to guide specific investigations. Because of the breadth of detection and sensitivity of these studies their findings can be used to generate new hypothesis to be tested in additional sets of experiments. This snapshot of the current context of transcriptomic studies indicate that there are some genes and pathways which affect several functions across different preparations (synaptic, glial and mitochondrial function) and represent key components to pathophysiological state of the typical (mutant *MECP2*) RTT patients. Such analyses can be used to uncover the biological basis of RTT and to point at new strategies for interventions.

## Abbreviations

AKT1: AKT Serine/Threonine Kinase 1
AMPA1: Glutamate Ionotropic Receptor AMPA Type Subunit 1
AMPA2: Glutamate Ionotropic Receptor AMPA Type Subunit 2
APLP1: Amyloid-Like Protein 1
ATP: Adenosine Tri-Phosphate
BA: Brodmann Areas
BDNF: Brain Derived Neurotrophic Factor
C1QA: Complement C1q A Chain
C1QB: Complement C1q B Chain
C1QC: Complement C1q C Chain
C3: Complement C3
CDKl5: Cyclin Dependent Kinase Like 5
CLU/APO-J: Clusterin
CNS: Central Nervous System
COX: Cytochrome C Oxidase
CRPM1: Collapsin Response Mediator Protein 1
CRYAB: Crystallin A Beta
CTD: C-Terminal Domain
CXCR1: C-X-C Motif Chemokine Receptor 1
DGE: Differential Gene Expression
DMN1: Dynamin 1
DOC2A: Double C2 Domain Alpha
EAAT1: Solute Carrier Family 1 Member 3
EAAT2: Solute Carrier Family 1 Member 2
FC: Frontal Cortex
FOXG1: Forkhead Box G
FXYD1: FXYD Domain Containing Ion Transport Regulator 1
GABRB3: Gamma-Aminobutyric Acid Type A Receptor Beta 3
GFAP: Glial Fibrillary Acidic Protein
HDAC: Histone Deacetylase 1
ID: Inter Domain
IL1: Interluekine-1 β
IL1R1: Interleukin 1 Receptor Type 1
IL-4: Interleukin 4
IPS: Induced Pluripotent Stem Cells
KEGG: Kyoto Encyclodpedia of Genes and Genomes
KLF4: Kruppel-Like Factor 4
KO: Knock-Out
KPNA3: Karyopherin Subunit Alpha 3
KPNA4: Karyopherin Subunit Alpha 4
MAP 2: Microtubule Associated Protein 2
MBD: Methyl Binding Domain.
MECP2: Methyl-CpG binding protein 2.
MRPS33: Mitochondrial Ribosomal Protein S33.
MT-CO1: Cytochrome C Oxidase.
MYC: MYC Proto-Oncogene BHLH Transcription Factor.
NFAT: Nuclear Factor Activated T-Cells.
NFATC3: Nuclear Factor of Activated T Cells 3.
NFκB: Nuclear Factor Kappa B Subunit 1.
NLS: Nuclear Localisation Signal.
NOTCH1: Notch Homolog 1 Translocation Associated.
NQO1: NADPH Quinone Oxidoreductase 1.
NR3C1: Nuclear Receptor subfamily 3 Group C member 1.
OCT4: Octamer binding Transcription Factor 4.
PBMC: Peripheral Blood Lymphomonocytes.
PKO: Peripheral *Mecp2* Knockout.
PMB: Post-Mortem Brain.
ROS: Reactive Oxygen Species.
RTT: Rett Syndrome.
S100A13: S100 Calcium Binding Protein A13.
S100β: S100 Calcium Binding Protein B.
SFG: Superior Frontal Gyrus.
SNAP25: Synaptosome Associated Protein 25.
SOX2: Sex Determining Region Y.
TGFBR2: Transforming Growth Factor Beta Receptor 2.
TGFβ: Transfroming Growth Factor Beta 1.
TRD: Transcriptional Repressor Domain.
TYROBP: TRYO Protein Tyrosine Kinase Binding Protein.
UPS: Ubiquitin Proteasome System.
UQCRC1: Cytochrome b-c 1 complex subunit 1.
Uqcrc1: Ubiquinol-cytochrome c reductase core protein 1.
XCI: X-Chromosome Inactivation.
